# Lay Health Worker Intervention Improved Compliance with Hepatitis B Vaccination in Asian Americans: Randomized Controlled Trial

**DOI:** 10.1371/journal.pone.0162683

**Published:** 2016-09-12

**Authors:** Hee-Soon Juon, Carol Strong, Frederic Kim, Eunmi Park, Sunmin Lee

**Affiliations:** 1 Department of Medical Oncology, Thomas Jefferson University, Philadelphia, Pennsylvania, United States of America; 2 Department of Public Health, National Cheng Kung University Hospital, College of Medicine, National Cheng Kung University, Tainan, Taiwan; 3 School of Medicine, Johns Hopkins University, Baltimore, Maryland, United States of America; 4 Department of Epidemiology and Biostatistics, School of Public Health, University of Maryland, College Park, Maryland, United States of America; The Chinese University of Hong Kong, HONG KONG

## Abstract

**Background:**

This study aimed to evaluate the effect of a lay health worker (LHW) telephone intervention on completing a series of hepatitis B virus (HBV) vaccinations among foreign-born Asian Americans in the Baltimore-Washington Metropolitan area.

**Methods:**

During the period of April 2013 and March 2014, we recruited Asian Americans who were 18 years of age and older in the community-based organizations. Of the 645 eligible participants, 600 (201 Chinese, 198 Korean, 201 Vietnamese) completed a pretest survey and received hepatitis B screening. Based on the screening results, we conducted a randomized controlled trial among those unprotected (HBsAg-/HBsAB-) by assigning them either to an intervention group (n = 124) or control group (n = 108). The intervention group received a list of resources by mails for where to get free vaccinations as well as reminder calls for vaccinations from trained LHWs, while the control group received only list of resources by mail. Seven months after mailing the HBV screening results, trained LHWs followed up with all participants by phone to ask how many of the recommended series of 3 vaccinations they had received: none, 1 or 2, or all 3 (complete). Their self-reported vaccinations were verified with the medical records. Multinomial logistic regressions were used to examine the effect of the LHW intervention. Process evaluation was conducted by asking study participants in the intervention group to evaluate the performance of the LHWs.

**Results:**

After seven months, those in the intervention group were more likely to have 1 or more vaccines than the control group, compared to the no vaccination group (OR = 3.04, 95% CI, 1.16, 8.00). Also, those in the intervention group were more likely to complete a series of vaccinations than the control group, compared to the no vaccination group (OR = 7.29, 95% CI 3.39, 15.67). The most important barrier preventing them from seeking hepatitis B vaccinations was lack of time to get the vaccination. The most important promoters to getting vaccinations, among those who had vaccinations (n = 89), were our intervention program (70.8%) and self-motivation (49.4%). The majority of participants in the intervention group received the phone calls from LHWs (93%) and almost all of them got the reminder to receive vaccines (98%).

**Conclusion:**

The LHW intervention was successful at increasing HBV vaccinations rates among foreign-born Asian Americans. This study suggests that this culturally integrated intervention program may be useful for reducing liver cancer disparities from chronic HBV infection in high risk Asian Americans.

**Trial Registration:**

ClinicalTrials.gov NCT02760537

## Introduction

Hepatitis B virus (HBV) infection has become a serious health concern within the Asian American community. While the prevalence of HBV infection is very low among Whites in the United States (0.1%), as many as 1 in 10 Asian Americans carry the virus, accounting for more than half of U.S. HBV cases [1]. About 65% of infected adults are unaware of their condition because chronic HBV is asymptomatic [2], yet the silent killer increases the risk of developing serious liver disease later in life. Because HBV disproportionately infects Asian Americans, significant liver cancer disparities exist among them, and they are often diagnosed with late-stage liver cancer, resulting in high mortality rates.

People who are not infected with HBV and who do not possess the HBV antibody should receive a series of 3 vaccinations in 6 months to protect them from the virus [2]. Most importantly, studies have shown that the hepatitis B vaccine as the first anticancer vaccine can protect them from hepatocellular carcinoma (HCC) [3]. Current U.S. policy is universal vaccination of all infants at birth, adolescents, and high-risk adults, such as intravenous drug users or those in close contact with HBV-infected individuals. In its most recent updated recommendation in 2009, the United States Preventive Services Task Force (USPSTF) advised that HBV vaccination is effective at lowering infection risk in high-risk populations [4]. While the cost effectiveness of HBV screening among the general population is under debate [5,6], HBV vaccination among foreign born adult immigrants is considered to be vital. A cost-effective analysis comparing several HBV screening and vaccination strategies among Asian and Pacific Islander adults found a screen, treat, and ring vaccination strategy was highly cost effective [7]. In this approach, everyone was screened by hepatitis B surface antigen (HBsAg) tests to determine whether they were chronically infected, and people with close contact with infected individuals were given hepatitis B surface antibody (HBsAb) tests and vaccinated if needed. However, as indicated in several community-based studies, the vaccination rate remained low among Asian American adults [8–11]. More than half of Asian American primary care providers reported less than a quarter of their adult Asian patients had received the HBV vaccination series [12].

Challenges exist in terms of properly informing and educating Asian Americans about HBV screening and vaccination. Therefore, culturally integrated educational strategies are needed to assure that we reach as many Asian Americans as possible. In previous studies, we developed a culturally integrated liver cancer educational program and tested the effectiveness of an intervention program for HBV screening using a cluster randomized controlled design. We found that participants in the intervention group significantly increased their HBV knowledge and were more likely to seek HBV screening compared to the control group [13,14].

The Asian American population has been growing rapidly in the United States over the past decade, and a majority (67%) of the current Asian American population is foreign born [15]. In 2010, Asian Americans numbered approximately 14.4 million, accounting for about 5.0% of the U.S. population, and by the year 2050, the Asian American population will be 41 million, accounting for 11% of the U.S. population, based on projected figures [16]. To ensure that Asian Americans receive needed hepatitis B care, it is important to fill knowledge gaps regarding HBV, enhance understanding of risks, and provide appropriate screening and vaccination.

Some studies have addressed ways to prevent HBV infection through various intervention programs [13,14,17–19]. Intervention programs educate high-risk populations about how HBV spreads, how to protect against the disease, and how to get proper care. Among these intervention programs, the lay health worker (LHW) model responds to the need for effective cancer interventions that reach at-risk underserved populations where traditional outreach efforts have failed [20,21]. A 2002 Institute of Medicine report on racial and ethnic disparities in healthcare recommended using LHWs as well as implementing collaborative interventions and preventive care programs [22]. In the past decade, more studies have examined the benefits of LHW models to raise hepatitis B awareness among Asian American communities. For example, a LHW intervention was found to be effective in increasing hepatitis B screening and knowledge among Hmong Americans [23] and Cambodian Americans [17] living in California. A similar study with Chinese Americans found that LHW intervention raised hepatitis B screening knowledge, but it had a very limited impact on screening test actions [19]. Most LHW studies conducted among Asian American have focused on increasing hepatitis B knowledge and self-reported screening, but very few have discussed the issue of completion of hepatitis B vaccinations. This gap in the HBV vaccinations among Asian Americans needs to be filled and evaluated. Also, despite evidence that the LHW interventions improve community outcomes of HBV screening behaviors among Asian Americans, few studies have reported process evaluations for monitoring the implementation of LHW intervention programs.

To address the disparity in hepatitis B care among the Asian American groups, intervention programs must consider barriers. Reasons for low levels of hepatitis B screening and vaccination among Asian American adults include low awareness and risk perception, a lack of access to health care because of limited insurance coverage and English proficiency [11,24,25], and cultural biases, such as believing that the consumption of Chinese herbal medicine will prevent acquiring HBV infection [26]. We intend to address these barriers in our LHW interventions and provide access to free or low-cost hepatitis B vaccinations. We aim to use a culturally-appropriate intervention that will prevent these barriers from becoming rampant. To address the gaps in the evidence, we undertook a study to examine the effectiveness of the LHW phone intervention on completing the series of HBV vaccinations and to identify the promoters to receiving vaccinations and the barriers preventing vaccinations. We hypothesize that those in the intervention group are more likely to complete vaccinations than those in the control group.

## Materials and Methods

### Study Design and Participants

This paper is part of a parent study of Asian American individuals who were found to be unprotected (n = 232) after a free hepatitis B screening. A randomized controlled trial (RCT) design was used to test the effectiveness of LHW interventions on adherence to vaccinations among those who were unprotected. The study period for the pretest/education program was between April 2013 and March 2014 and for the follow-up was between January 2014 and February 2015. This study was approved by the Committee on Human Research of Johns Hopkins Bloomberg School of Public Health in March 2013. There was a delay in registering the trial as we were made aware of the benefits of registering the trial after data collection had begun. No changes were made to the study protocol between the start of the trial and the time of registration. The authors confirm that all ongoing and related trials for this intervention are registered at ClinicalTrials.gov (NCT02760537).

In the parent study for the Asian American Liver Cancer Prevention Program (hereinafter the Program), foreign-born Asian American adults, aged 18 years and older, and never had hepatitis B testing, were recruited from the community-based organizations in the Baltimore-Washington Metropolitan Area using a non-probability sampling. One or two weeks before the Program, we had a pre-screening event to recruit eligible participants. Of the 645 eligible volunteer participants, 30 did not show up for the program. Of the 615 who came to the program, 15 did not complete either survey or hepatitis B screening. A total of 600 completed the survey and screening. Among those 600 screened participants (201 Chinese, 198 Korean, and 201 Vietnamese immigrants), 33 (5.5%) had chronic HBV infection and 335 (55.8%) had evidence of resolved HBV infection (protected). A total of 232 (38.7%) were susceptible to HBV infection (unprotected).

**Fig 1:**
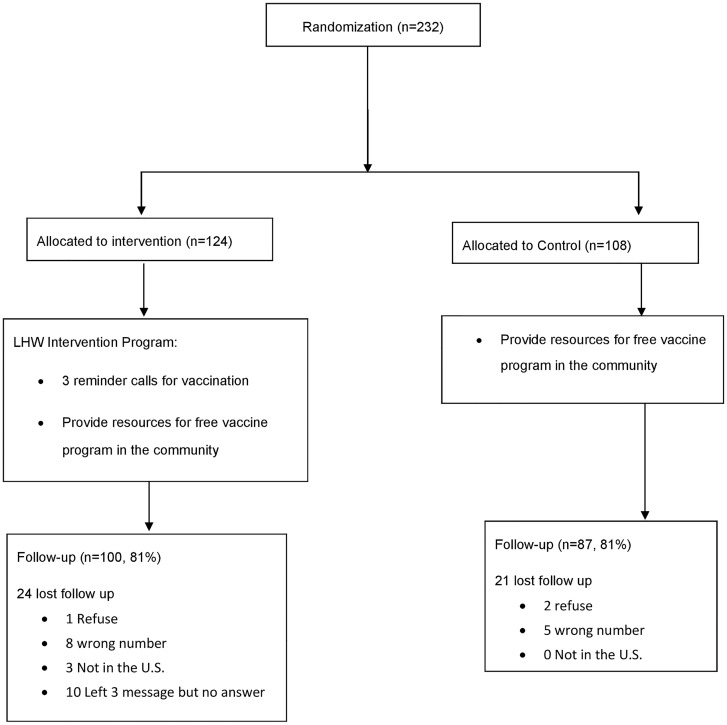
Flow of participants through randomized trial.

Fig 1 summarizes the study design. Of the 232 unprotected, 124 (53.4%) were assigned to the intervention group and 108 (46.6%) were assigned to the control group. 185 (79.7%) completed 7-month follow-up. Among the 47 (20.3%) who did not follow-up, 2 did not complete the posttest (partial completion) and 3 refused to be followed up; 13 had a wrong phone number or phone disconnected; 3 were not in the United States; 21 did not answer the phone after 3 voice messages; 5 did not set up voice mail and could not be reached (See Fig 1).

### Recruitment procedures

To obtain the referred recruitment locations, we used long-term connections (since 2008) with local community-based organizations (CBOs) through the community advisory board. The three major recruitment strategies adopted for participants were: (1) Advertisements describing the study were placed in local Chinese, Korean, and Vietnamese newspapers and in local Asian grocery stores, and those who called were screened for eligibility and invited to the study; (2) Community announcements of upcoming screening events by LHWs, who were trained about hepatitis B by members of the research team during a 7-hour all-day program; and (3) Contact with church and temple leaders to arrange recruitment days where church and temple members were recruited to participate in the study on the spot. Most participants were recruited directly by LHWs or learned about screening events through word-of-mouth.

### Data collection procedures

#### Pre-test/Education program

After providing written informed consent, all of the participants were asked to complete a self-administered questionnaire in English, Chinese, Korean, or Vietnamese with the assistance of a bilingual interviewer when necessary. Then, all of the participants were instructed and given 5 to 10 to minutes to read culturally integrated and linguistically appropriate educational materials (e.g., a photonovels) that had been developed and validated for efficacy from prior studies [13,14,27].

#### HBV screening test/Informing the results

All participants received hepatitis B testing for HBsAg, HBsAb, and HBcAb (hepatitis B core antibody). A week later, they received the results of the screening test. Based on the screening results, all participants were categorized into three groups: (1) infected (HbsAg+), (2) unprotected (HbsAg-/HbsAb-), or (3) protected (HbsAg-/HbsAb+). We sent the results by mail to participants who were unprotected and protected. We did not follow up with healthy participants who were protected. A medical doctor followed up with those infected with a phone call to explain the results and medical counseling.

#### Lay health worker recruitment and training

LHWs were recruited from each of the ethnic communities (i.e., Chinese, Korean, and Vietnamese) within the collaborating CBOs in the Baltimore/Washington Metropolitan area. The recruitment process included the distribution of flyers to the CBOs, the release of information through CBO e-mail lists, the distribution of flyers throughout university campuses, and the publishing of information on CBO websites. The LHW was required to be 21 years or older and a bilingual speaker of English and Mandarin, Vietnamese, or Korean. Training sessions, each of 7 hours in duration, occurred during December 2012 and December 2013. A total of 89 LHWs participated in 5 training sessions that each contained 2 phases: the first phase offered an interactive education to targeted Asian American communities and the second phase provided training for delivery of telephone-based interventions.

The training for the LHWs comprised the following core competencies: (1) basic public health information about liver cancer prevention and available options for treatment in local areas (core competency: knowledge of prevention and treatment); (2) the ability to communicate with community members about the importance of hepatitis B screening and vaccination, and to recruit them to participate in screening events (core competency: identifying community contacts); (3) providing community education to increase awareness of liver cancer prevention strategies (core competency: teaching); and (4) reminding community members about follow-up actions, such as screening and vaccination (core competency: coaching and navigation assistance). These training sessions implemented PowerPoint presentations, role-playing videos, photo novel brochures, and group discussions in each LHW’s native language. To facilitate their education efforts within their respective communities, LHWs were provided with either a paper copy or electronic access to the training materials, including the PowerPoint presentations, role-playing videos, and photo novel brochures. LHWs needed to complete the training in order to be qualified to conduct the phone intervention in the community.

#### LHW intervention for those unprotected

Those unprotected (n = 232) were randomly assigned to either the intervention (n = 124) or the control (n = 108) groups by computer-automated random assignment (1:1). If two family members participated in the study with the same result of unprotected (n = 15), we ensured that both were assigned to the same group to prevent contamination. Randomization was used to ensure equivalence between groups on key factors that may potentially influence the outcome of HBV vaccinations: gender, age, education, length of stay in the United States. The software eliminated the need to do a stratified sampling since it randomly dispersed participants with equivalent levels across two groups.

Among those assigned to the intervention group, LHWs conducted phone interventions by reminding participants of a series of vaccinations at months 1, 2, and 5. Those who had health insurance were encouraged to complete vaccinations through their providers. For those who did not have health insurance, LHWs helped them access vaccinations by referring them to free vaccine events in the community. Those in the control group received their results by mail, along with a list of resources that offered free vaccinations, such as local health departments.

#### Endpoint Survey and Medical Verification Card

Seven months after mailing the results, the LHWs followed up with those found unprotected by telephoning to ask about the status of their series of vaccinations and about promoters or barriers to vaccinations. Those in the intervention group were also asked about their experiences interacting with the LHWs. Their self-reported vaccinations were verified with the medical records. They were asked to provide information about the date of vaccinations as well as the location of the clinic or doctor’s office where they received vaccinations. They were also asked to sign a medical release form giving project staff permission to request medical records regarding their vaccinations. Each participant was given a stamped vaccination card to fill out the date and provide the signature of their physician following each completed hepatitis B vaccination. Participants were asked to mail the card back to the research team once the card was completed.

#### Process Evaluation

We evaluated the performance of the LHWs interviewing by phone those in the intervention group who had interacted with LHWs during the 7-month follow-up. We focused on whether they had received vaccination reminder phone calls and assistance from LHWs. Intervention participants were also asked to rate their LHW’s level of expertise on HBV-related subjects such as whether they considered the LHWs to be knowledgeable and helpful, whether they posed questions to the LHWs, whether they felt comfortable posing questions, and whether the LHWs answered their questions.

#### Sample Attrition

About 80% of the participants completed the phone survey after the 7-month follow-up. Those who dropped out of the study (n = 45, attrition rate = 19.4%) were not statistically different from those who followed up in terms of age, gender, education, ethnicity, employment, health insurance status, family history of Hepatitis B infection, spoken-English proficiency, self-rated health, or knowledge of HBV transmission (Table not shown). However, marital status was associated with the drop-out: those who were married were more likely to be followed-up than those who were not married (83.3% vs. 64.4%, p<0.05).

### Measures

The outcome measure was whether participants had received a series of vaccines over 6 months. Receipt of vaccines was divided into 3 categories: no vaccinations (= 0), 1 or 2 vaccinations (= 1), or a series of 3 vaccinations (= 2, completion of HBV vaccination).

The following information on sociodemographic characteristics and family history of HBV infection at baseline were included: age, gender, race/ethnicity, education, employment, marital status, health insurance status, spoken-English proficiency, and family history of HBV infection. For knowledge scores of HBV transmission mode, participants were asked 10 questions on how HBV can be spread (e.g., from infected mother to child during child birth, by sharing a toothbrush with an infected person, by having sexual intercourse with an infected person). Each correctly answered question was given a point (= 1), and the sum of the scores ranged from 0 to 10 [14].

### Statistical analysis

This 7-month RCT assessed the effectiveness on HBV vaccination completion of the LHW reminder call intervention. Since our main hypothesis was that the intervention group was more likely to complete a series of vaccinations compared to the control group, we conducted multivariate multinomial logistic regression with 3 categories of outcome: none, 1 or 2, or all 3 (complete). We, then, conducted a subgroup analysis to examine the promoters to vaccinations among those who had vaccinations and the barriers to vaccinations among those who did not have vaccinations at all.

## Results

Baseline characteristics for each group are presented in Table 1. The average age of the 232 respondents was 48.8 years; Ethnicity was evenly distributed (32% of Chinese and 34% of Koreans and Vietnamese). 56.9% were females; about 13% had less than high school education. About two thirds were employed. About half had health insurance. About 12% reported that they had a family history of HBV infection. One third rated their spoken-English proficiency as poor or not at all. About 40% reported their health status as fair or poor.

**Table 1:** Baseline characteristics in the Baltimore Washington Metropolitan Area, 2013–2014 (n = 232).

	Total (n = 232)	Intervention (n = 124)	Control (n = 108)
	N(%)		
**Age (years), mean (SD)**	48.8 (.76)	49.3(.98)	48.2(1.18)
**Gender**			
Men	100 (43.1)	45.2	44
Women	132 (56.9)	54.8	48.5
**Ethnicity**			
Korean	79 (34.1)	33.9	34.3
Vietnamese	78 (33.6)	30.7	37
Chinese	75 (32.3)	35.5	28.7
**Education**			
<High school	31 (13.4)	14.5	12.2
High school Plus	85 (36.8)	34.7	29.3
College graduate	67 (29.0)	26.6	31.8
Grad school	48 (20.8)	24.2	16.8
**Employment**			
Employed	157 (67.9)	65.3	71
Not employed	74 (32.1)	34.7	29
**Having health insurance**			
Yes	212 (52.8)	55.3	50
No	108 (47.2)	44.7	50
**Family history of hepatitis B infection**			
Yes	27 (11.6)	15.3	7.4
No	134 (57.8)	53.2	63
Not sure	71 (30.6)	31.9	29.6
**Spoken English proficiency**			
Fluent/well	58 (25.0)	21.8	28.7
So so	93 (40.1)	42.7	37
Poorly/not at all	91 (34.9)	35.5	34.3
**Self-rated physical health**			
Excellent/very good	67 (28.9)	24.2	34.3
Good	80 (34.5)	32.3	37
Fair/poor	85 (36.6)	43.5	34.3
**Knowledge of HBV transmission mode (0–10), mean (SD)**	4.4 (2.6)	4.2 (2.6)	4.5 (2.6)

Note. SD stands for standard deviation.

### Medical record verification

Table 2 shows differences between self-report of vaccinations and medical verification cards. Of 187 who completed follow-up, 66 (n = 35.3%) participants self-reported that they had completed a series of 3 HBV vaccinations in the past 6 months. We were able to receive medical verification cards for 65 of 187 (excluding the 3 participants did not participate in posttest and returned the medical verification card). About 89% (58/65) reported that they completed the series of vaccinations and sent the medical verification card. The agreement between self-report and medical verification card was substantial using the kappa statistic to correct chance (kappa = .82).

**Table 2:** Agreement between self-report and medical verification cards for completing a series of vaccinations (n = 187).

	Medical verification card		
Self-report	No (%)	Yes (%)	total
**No**	114 (93.4)	7 (10.8)	121
**Yes**	8 (5.6)	58 (89.2)	66
**total**	122	65	187

Note. Kappa = .82.

Table 3 compares the percentage of participants in the intervention (n = 100) and control groups (n = 87) who had HBV vaccinations. Those in the intervention group were more likely to complete a series of 3 vaccinations than the control group (51% vs. 15%). There was little difference between the intervention group and control group in terms of receiving 1 or 2 vaccinations (15% vs. 10%). Finally, those in the intervention group were more likely to complete vaccinations than those in the control group (62% vs. 34%). Chi-square testing showed that these results were significant (p = 0.001).

**Table 3:** HBV vaccinations among Asian Americans Randomized to intervention and control groups using Lay Health Workers: Baltimore Washington Metropolitan Area, 2013–2014 (n = 187).

Number of vaccinations	Control group, No (%)	Intervention group, No (%)	OR (95% CI^a^)
No vaccination	62 (71)	34 (34)	1.00
Had 1 or 2 vaccinations	10 (12)	15 (15)	3.04 (1.16, 8.00)
Completed a series of 3 vaccinations	15 (17)	51 (51)	7.29 (3.39, 15.67)
Total	87	100	

Note. Chi-square statistic = 28.04 (2df, p = <.0001);

^a^CI = confidence interval.

In the multinomial regression, we examined the effect of LHW intervention on vaccinations (see Table 3). Those in the intervention group were about three times more likely to have 1 or more vaccines than the control group (OR = 3.04, 95% CI 1.16, 8.00) compared to those who had never received a hepatitis B vaccination. Those in the intervention group were seven times more likely to complete a series of vaccinations than those in the control group (OR = 7.29, 95% CI 3.39, 5.67) compared to those who never received a vaccination.

### Promoters to HBV vaccinations

Several factors motivated participants to receive hepatitis B vaccinations in the 7 months following the free screening. Among the 89 respondents who obtained vaccinations in that period (2 did not complete a posttest), 70.8% (n = 63) reported our screening program and educational program (e.g., reading photo novels) motivated them to do so. About half (49.4%) reported that they were motivated by self-awareness after receiving the letter along with screening results.

### Barriers to HBV vaccinations

We explored the barriers to receiving HBV vaccinations among those who had received no vaccinations. Out of the sample of 96, 46% indicated that they did not have time; 19% did not know where to get the vaccination; 13% either had no health insurance or could not afford to get a vaccination; 11% reported that receiving the vaccination was not important to them; 9% forgot to receive the vaccination.

### Process evaluation

In Table 4, we evaluated the performance of the LHWs among those in the intervention group (n = 100). The majority of intervention participants had received phone calls from LHWs (93%) and almost all of them got the reminder to receive vaccines (98%). They LHWs also provided additional information, such as resources to vaccination (44%) and clarification of results (15%). Participants had a chance to ask questions if they had any (62%), and regardless of whether they actually asked questions, 94% said they felt comfortable asking questions. When participants were asked to rate LHWs’ level of expertise on HBV-related subject, most people responded positively, such as stating that they considered their LHW to be very knowledgeable (46%) or somewhat knowledgeable (29%), and very helpful (63%) or helpful (31%).

**Table 4:** Participants’ evaluation of LHW intervention among those in the intervention group (n = 100).

Content	Options	N	%
Received LHW call	Yes	92	92.9
	No	7	7.1
Information provided (total n = 92)	Clarification of results	14	15.2
	Reminder to receive vaccines	90	97.8
	Resources to vaccination	40	43.5
	General info about Hepatitis B	2	2.2
Easy to understand information (total n = 90)	Yes	75	81.5
Asked the questions (total n = 90)	Yes	56	62.2
Comfortable to ask questions (total n = 90)	Yes	85	94.4
LHW answered questions (total n = 90)	Yes	64	71.1
Feel LHW			
Knowledgeable (total n = 89)	Very Knowledgeable	41	46.1
	Somewhat knowledgeable	26	29.2
	Not quite knowledgeable	6	6.7
	Not knowledgeable at all	2	2.2
	Not applicable	14	15.7
Helpful (total n = 90)	Very Helpful	57	63.3
	Helpful	28	31.1
	Slightly helpful	3	3.3
	Not helpful at all	2	2.2
Overall evaluation (total n = 90)	Liked very much	63	70
	Liked it	21	23.3
	So-so	5	5.6
	Did not like	1	1.1

## Discussion

The results from our LHW intervention program to encourage Asian Americans to receive HBV vaccinations give us insights into what factors motivate people to take the proper steps to complete HBV vaccinations. With this program implementation, we saw more Asian Americans becoming educated about HBV and taking appropriate actions to seek prevention. Our intervention program increased the likelihood that those in the intervention group would receive HBV vaccines than those in the control group.

A salient issue for having HBV vaccinations during the 7-month follow-up period was whether participants were motivated by our program. We learned that our educational program (e.g., reading photo novels) was one of the most important promoters to motivate them to do so. Both intervention and control group encouraged to read our culturally and linguistically developed educational material after initial survey and before free screening. The other promoter is self-awareness after receiving the letter along with screening results. This result suggests that photonovels are effective teaching tools, especially for low literacy foreign-born Asian Americans.

When we consider the barriers to receiving vaccinations, we noted that time was one of the main barriers. About half of the participants stated that they did not have time to get vaccinations since they had very busy lives, such as working with long hours and taking care of a family. One out of five participants also said that they did not receive vaccinations because they did not know where to get them. This barrier could be easily remedied by ensuring that the information for about vaccine resources is clearly stated and readily available. Participants also reported that they did not receive vaccinations because they had no health insurance or could not afford to get a vaccination. Cost of hepatitis B vaccination may no longer be a major barrier since the Affordable Care Act (ACA) now requires coverage of a few recommended preventive services, including hepatitis B vaccination, without cost sharing for non-grandfathered private health insurance plans, Medicare, and some Medicaid plans [28]. Interventions such as our LHW intervention are important because they can provide knowledge and reminders about hepatitis B that can spur community members to access these free vaccinations.

Our study uniquely contributes to improving the hepatitis B vaccination rate among Asian Americans by addressing specific barriers in this population. Very few prior studies have addressed such issues and even fewer have provided evidence of process evaluation [29]. Even with free vaccinations provided, the completion of all three shots of vaccination can still be lower than expected [30]. Because LHWs are people who know their assigned community well, it would be advantageous to investigate how they could share not just their knowledge of HBV but also their knowledge of vaccination resources with their community. Each ethnic community is different in its own way in terms of the kinds of community organizations currently active in different regions and the kinds of outreach activities that are effective. Gathering such information could be conducted in future studies through focus groups with the LHWs after the training. Not only could information gathering share potential approaches in how to spread the information around the community, but also it could assist the investigators to identify the communication channels within each ethnic community and how the knowledge could be further distributed widely in the Asian American communities.

This study had several limitations. A sampling frame from CBOs was used, but to minimize the sampling bias due to excluding those who do not participate in these organizations, we tried alternative ways to recruit participants by going to health fairs and ethnic shopping centers. The majority of the sample, however, came from the CBOs, so the study cannot be generalized to other Asian Americans who do not attend any of the CBOs. Second, we also have to consider the possibility of self-selection bias since most participated in this study based on their interests

This study also had considerable strengths. Our study differs from previous studies of self-reported HBV screening or vaccinations in the Asian American population [19,23, 31–34]. We provided all the participants free HBV screening at baseline to identify their status of HBV infection without any recall bias of self-report, minimizing potential errors in reporting HBV status. Second, our LHW intervention program increased the completion of a series of vaccinations by reminding those unprotected of the importance of vaccinations and time to have vaccinations and by providing them resources for free vaccine events in the community in the targeted area. We also addressed several critical components for the process evaluation of an LHW phone intervention with Asian Americans and found that trained LHWs were knowledgeable and provided follow-up action making reminder calls, as indicated by evaluations from intervention participants. In addition, LHW intervention provides sustainability of intervention as a community-owned program by implementing LHWs. Third, using medical record verification, we checked the consistency between self-reports of vaccination and medical verification cards which turned out to be very high. Finally, this study is the collaborative work between academic institutions and CBOs committed to outreaching high-risk population for HBV screening and to connecting participants with free vaccine events organized by Hepatitis B Initiative-DC (HBI-DC), a CBO that aims to mobilize communities to prevent liver diseases among communities at risk in the Washington, D.C. metropolitan area.

## Conclusion

This LHW telephone intervention program yielded a substantial increase in the likelihood of vaccinations in underserved, high-risk, Asian minority populations. It indicates that LWH intervention method should be disseminated in a large sample of high-risk of Asian Americans to contribute to reducing liver cancer disparities. We recommended several additional ways to ensure that more Asian Americans to increase compliance of hepatitis B vaccinations and to spread the word of the importance of protecting against liver cancer. For example, social media, mass e-mailing lists, or mobile health interventions are techniques that could be utilized to reach a more diverse Asian American population. In the context of the coverage of hepatitis B vaccination services under ACA, we encourage the LHW intervention demonstrated in this paper to be disseminated in various Asian American communities throughout the United States. With the availability of LHW training material and resources in various Asian languages on the internet for free download, such as photo novel brochures and role-playing videos, this LHW intervention program should be easily implemented.

## Supporting Information

S1 Checklist CONSORT 2010 Checklist. (DOC)

S1 Data Stata dataset for the manscript. (DTA)

S1 IRB IRB application. (PDF)
