# Supplementary material for: Lay Health Worker Intervention Improved Compliance with Hepatitis B Vaccination in Asian Americans: Randomized Controlled Trial
Source: PLoS One. 2016 Sep 12;11(9):e0162683. doi: 10.1371/journal.pone.0162683 (PMC5019387; doi:10.1371/journal.pone.0162683)
Supplement: S1 IRB — (PDF) [file pone.0162683.s003.pdf]

RESEARCH PLAN

PI: Hee-Soon Juon
Study Title: Lay Health Worker Model to Reduce Liver Cancer Disparities in Asian Americans
IRB No.: 00004537
Date: January 31, 2013

1. Aims/Objectives/Research Question/hypotheses:
The objective of this project is to build a sustainable, community-based, participatory program of lay health workers (LHWs) to promote liver cancer prevention programs among high-risk groups of Asian Americans. The goal of this study is to implement culturally integrated liver cancer prevention programs for Chinese, Korean, and Vietnamese-Americans (CKV-As) through outreach, screening, education, research, and training in the Baltimore Washington Metropolitan Area. Given these broad objectives, the proposed program will achieve the following specific aims:

1. Develop a training protocol and certificate program for LHWs to conduct sustainable, community-based liver cancer prevention programs;
2. Assess the prevalence of HBV infection by providing hepatitis B testing among Chinese, Korean, and Vietnamese-Americans (CKV-As);
3. Implement intervention programs, based on screening results
   a. For those unprotected, provide LHW intervention programs on their adherence to HBV vaccinations using Randomized Controlled Trial;
   b. For those infected, provide patient-navigation services for antiviral therapy and follow-up for the treatment;
   c. For those infected, follow up annual blood testing/banking to build a database for a future cohort study or case-control study
4. Evaluate the effectiveness of LHW intervention on HBV vaccinations among those unprotected.

2. Background and Rationale:

Hepatitis B—the world's most common serious viral infection of the liver—can cause premature death from liver disease or liver cancer. Chronic hepatitis B and liver cancer caused by hepatitis B in Asian Americans and Pacific Islanders (AAPIs) comprise one of the most serious—but frequently neglected—racial and ethnic disparities in the U.S. (Office of Minority Health 2008). AAPIs comprise only 4% of the U.S. population, yet account for over half of the 1.25 million people with chronic hepatitis B virus (HBV) infection and half of the 5,000 Americans who died from liver failure resulting from chronic HBV infection from 1999 to 2002 (Margolis et al. 2006; Armstrong et al. 2006). About 65% of hepatitis-B infected adults do not find signs of symptoms by themselves. Infected mothers spread the virus to children at birth. In combination with cultural, linguistic, and financial barriers, this excess risk can be even more highly associated with higher HBV infection rates, particularly for risk groups, among foreign-born Asian immigrants. Delivering appropriate prevention and treatment services to these groups can reduce the societal and economic costs of these diseases. The CDC projects that each 1 million high-risk adults vaccinated would save up to $100 million in future, direct medical costs by preventing 50,000 new hepatitis B infections, 1,000-3,000 chronic hepatitis B infections, and 150-450 deaths from cirrhosis and liver cancer (CDC 2003). Because of the low rates of HBV screening and insufficient knowledge of liver cancer prevention, Asian Americans (AAs) are often diagnosed with late-stage cancer, resulting in low survival rates and high mortality rates. Compared to other racial/ethnic groups, Hepatitis B disproportionately infects AAs and thus leads to significant liver cancer disparities among AAs.

3. Develop a training protocol and certification program for LHWs
We will develop a LHW model that highlights the effects of culturally competent human and educational interactions on facilitating the CKV-A community's active behaviors in hepatitis B screening, vaccination, and

treatments. To develop an efficacious and effective LHW intervention that will contribute for creating a critical mass toward sustainable CKV changes, our strategic approaches include as follows:

Processes for Development of a CKV Community LHW Intervention Model

1. Review the liver cancer education program guidebook in English, (developed by ongoing project R25).
2. Refine/revise the approaches through work group reviews: Through a series of bi-monthly meetings with participants from CAB, CKV-A communities, health practitioners and outreach workers, and experts in HBV and liver cancer education (Juon and Lee), and education and evaluation (Park), we will confirm/specify the approaches of the available materials and develop additional assessment approaches and forms in English **(See the attachment for LHW training protocol).**
3. Establish the LHW model to certify satisfactory practicum experience in Patient Navigation training module: Through ongoing assessments with the study participants and participating health care systems, we will monitor the LHW experiential learning module to provide patient navigation function. The approach will identify barriers to hinder access to timely screening, follow-up, and treatment upon diagnosis.

Recruitment for LHWs: Earp et al. (1997) suggest effective LHWs would have some or all of the following characteristics and skills: (1) the ability to comfortably and effectively access the health care system; (2) the ability to balance the demands of lay helping with other life responsibilities; (3) an interest in AA's health and social issues; (4) maintain close, supportive, and reciprocal connections with others; and (5) leadership skills. LHW models depend on genuine natural helpers to function. To increase recruitment potential for CKVs having desired characteristics: First, we will reach a broad pool of adult volunteers, regardless of gender, income level, or health care experience, who have these characteristics and also have the ability to speak, read and write Chinese, Korean, or Vietnamese languages at or above 2nd grade level. Second, adopting a referral recruitment strategy in social market theory (Lefebvre et al. 1988) as it may be more effective for people who may be more responsive to their sense of collective cultural group's interactions (Hofstede 1991), we will recruit the first 10 LHW candidates per each ethnic group referred by word of mouth, faith-based organization leaders, community advisory groups, and health and social service agencies. For example, LHWs will be recruited from the community by referring from the president of CBOs who is the members of the Community Advisory Board for this project. We will also recruit LHWs by advertising in the ethnic media (e.g., newspaper, radio) and by putting fliers at the ethnic groceries. Finally, we will expand our recruitment continuously in the community. In Year 1, we plan to train a total of 60 certified LHWs (20 from each ethnic group). We estimate retention of 40 (67%) certified LHWs at the end of the Year 1, based upon literature reviews (Anderson et al. 2000). To replace drop-out LHWs in subsequent years, we plan to recruit and train a total of 30 additional LHWs (10 for each ethnic group) from Years 2 to 3.
Training for LHWs: PI and Investigators will train the bilingual staffs in English. Those trained staffs will provide the training session to LHWs in Chinese, Korean, and Vietnamese. Those LHWs will get paid $50 for their participation of one-day training session. Later, they will get paid another $50 for completing other tasks including recruitment of participants, community education, and telephone interventions (A copy of LHW agreement is uploaded).

Training: The metric highlights a logic model for outcome-oriented intervention approaches.

Aimed Primary Outcomes<br>As the LHW program will be implemented, the outreached community members (CM) can		Core Competence of LHWs<br>In order to facilitate the CM outcomes, a LHW can	Requirement for LHW Certificate of completion<br>The certificate of completion will be issued if LHW can demonstrate successfully the required evidence in Core Competence areas.	LHW Training Protocol<br>In order to recruit and develop more effective LHWs who can assist CM' sensitive issues,
Know basic public health knowledge in liver cancer prevention/control<br><br>Know available	←	-Know liver cancer and HepB related issues<br>-Conduct a 30-minute liver education program developed by R25<br>-Provide CMs available	Upon satisfactory completion of the one-day (7 hours) training workshop and experiential practicum (i.e. internship),<br>- Satisfactory knowledge test score (e.g. cut point at 80%	First, we will maximize the established networks of active community-based organizations (CBOs) in this region

resources for Hep B project<br>- photonovel<br>- LHW	resources regarding Hep B screening events, free vaccinations, and referral resources for treatment	correct)<br>- A questionnaire responded by teaching session participants	Second, we will refine the approaches in order to develop three culturally tested versions in CKV languages through the validity pilot tests.<br><br>Finally, all planned procedures and policies related to training will be updated through pilots with focus groups of CKVs prior to implement it.<br><br>The Education Committee will be responsible to set the standard in the Certificate of LHW Competence, and will review the internship/field practicum portfolio.<br><br>Upon satisfactory completion and approval of the LHW Performance Assurance Committee, Certificate of completion in Asian American Liver Cancer will be issued. Anyone who receives a below-satisfactory final evaluation will have unlimited opportunities to join the next available training cohorts.
Participate in the proposed study through<br>-Free HBV screening events organized by HBI-DC during the study period	-Recruit study participants at the free screening events and community<br>-Facilitate to prioritize the importance of screening and to avoid barriers	-Phone call logs<br>-Posting Flyers (logs for number and place)<br>-Field note included CMs' barriers and promoters to participate in the screening events<br>- A list of CM visitors who participated at the screening events.	
Follow-up action upon screening results:<br><br><u>Unprotected (50%)</u><br>Complete a series of vaccinations	Perform the role as a patient navigator for those unprotected<br><br>Conduct LHW intervention	- Receive field training for intervention (e.g., role, respect, voluntary participation, privacy, confidentiality)<br><br>- Field note included the CMs' special needs, concerns, inquires, solutions, etc.<br>- Reflection note on practicum experience<br><br>- The final portfolio collected supporting evidence in Core Competence. The completion of all requirements grants eligibility for certificate consideration.	

Process Evaluation: Using evaluation model of RE-AIM (Glasgow & Linnan 2008), we will examine the extent to which the LHW program is carried out through processes as intended. At individual level, we will focus on Reach (R) of the LHW certificate of completion program and on the Efficacy/Effectiveness (E) of the program. For the group level of aggregated individuals, we will assess on Adoption (A), Implementation (I), and Maintenance (M). We will conduct semi-structured interviews and/or surveys collecting through opportunities for hep B free screening events such as screening day, 3-month, 7-month, and annual follow-up assessments. In addition, we will assess general administrative processes of LHW program during the study period in the selected perspectives of fidelity (e.g. logistic reviews, completion rates), dose (e.g. satisfaction survey), and method on translated forms and tools (Saunders, Evans, & Joshi 2005).

Outcome evaluation: In 7 months follow-up for those unprotected, we will evaluate outcomes of LHW intervention in the following: 1) health outcomes (e.g., completion of a series of vaccinations); 2) participant satisfaction (e.g., who encourage to complete vaccinations; whether they receive the reminder; how they like the reminder call and more); 3) improvement in knowledge about or awareness of HBV infection and liver cancer.

4. Participants:

A total of 600 foreign-born Asian American adults (200 Chinese, Koreans, and Vietnamese each), 18 years of age and older will be drawn in the Baltimore Washington Metropolitan Area.

This population was chosen because: 1) they are at higher risk of hepatitis B virus (HBV) infection than any other ethnic groups, and 2) they are the population who report low adoption of HBV screening and vaccinations. We expect to have 600 participants during the study period. We expect the gender and ethnicity distribution in our study population to reflect the diversity and distribution in the targeted areas. The following inclusion and exclusion criteria will apply:

Inclusion Criteria:

Persons will be eligible for study if they: 1) are older than 18 years; 2) are self-identified foreign born individuals in China, Korea, and Vietnam; 3) will stay in the targeted area in the next 2 years; and 4) are not aware of HBV infection.

Exclusion Criteria:

Persons will be excluded if they are 1) unwilling or unable to give informed consent; 2) pregnant women; or 3) not a resident in the Baltimore Washington Metropolitan Area.

<u>Sample size estimate</u>: Based on our previous studies (Juon et al. 2008; Hsu et al. 2007), we estimate that about 10% of all the participants will test HBV positive, 40 to 50% will be HBV negative but protected, and about 50% will be unprotected. In the preliminary analysis of R25, there were significant mean differences of knowledge of HBV transmission (score ranged 0-10) in intervention (7.21±1.83SD) and control group (5.54±2.07SD) in post-test. We estimated our sample size to be 50 in each intervention and control group among those unprotected. Assuming a conservative rate of attrition about 20%, we calculated the study power based on sample size at follow-up. The table shows the sample size of 50 at pre-test is to detect mean difference in knowledge outcome at 96% power (1-β) and type I error of 0.05 (Cohen 1998).Thus, our proposed sample size (n=50) for each group among those unprotected has sufficient power. Our total sample size is 600 (50/group x 2 groups x 2 (infected and protected-50% of study population) x 3 ethnic groups).

Sample size at pre-test	Sample size at post-test	power (1-B)
40	32	.92
50	40	.96
60	48	.98

Identifiers will be collected for follow-up including name, address, telephone number, and e-mail address.

<u>5. Study procedures</u>:

1) Study design/methods

This is a 4-year longitudinal study of hepatitis B screening programs with randomized controlled trial (see the figure).

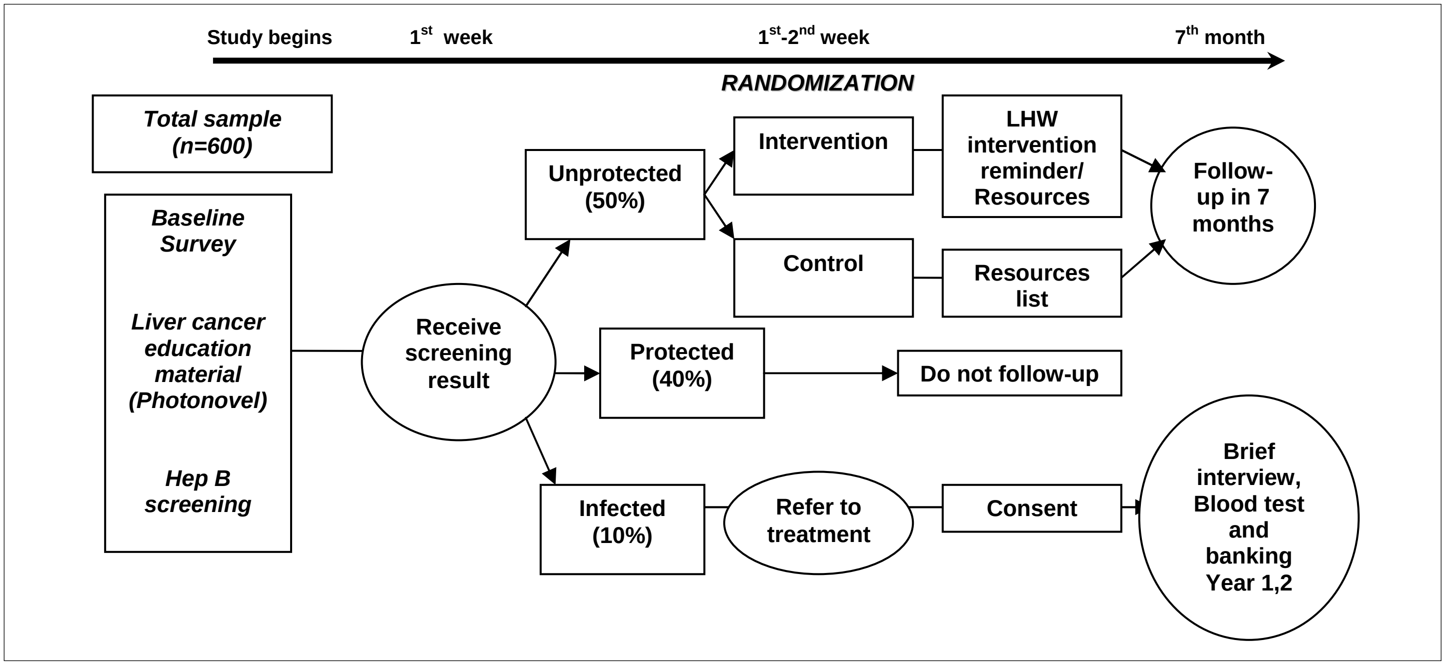

2) Data collection procedures

1. **Recruitment**: We have four major channels to recruit participants from the community: 1) Health Fair organized by HBI-DC; 2) AAHC volunteer clinics to recruit participants who have interests in our project (either patients or family members). We are not going to use their medical records in the clinic. AAHC provides a space for us to conduct the research project including survey, education, and HBV screening test. Those who will be recruited from the community will ask to come to AAHC to participate in our study; 3) LHWs' announcement of upcoming screening events in their community by advertising in Korean churches or Chinese language schools or by word-of-mouth; and 4) by advertising our project in ethnic media and ethnic groceries.
2. **Pre-test:** After obtaining the informed consent for screening test and blood banking, all the participants will be asked to complete a self-administered questionnaire in English, Chinese, Korean, or Vietnamese, with the assistance of a bilingual interviewer when necessary. Then, they will be asked to have anthropometric measures (e.g., height, weight, waist and hip circumference) as well as to check blood pressure.
3. **Education program:** All participants will receive a culturally integrated liver cancer educational material (e.g., photonovel) and ask them to read it. Then, they will ask questions if they like to clarify something they do not understand.
 HBV screening test/initial biospecimen banking: All participants will receive hepatitis B testing. For blood sampling procedure, we will have a part-time phlebotomist working in the field with a bilingual staff and LHW. The phlebotomist will draw two tubes of blood from study participants at the study site (e.g., AAHC, HBI-DC). He/she will then transport one tube of the blood in red-top tube to certified clinical labs (i.e., JHMI, Quest, LabCorp) for hepatitis B tests (e.g., HBsAg, HBsAb, HBcAb). We will send the blood for screening to the clinical lab:
 The Johns Hopkins Hospital, Department of Pathology,
 Carnegie 423, 600 N. Wolfe St., Baltimore, MD 21287
 (The hard copy of CLIA accreditation is uploaded). The Clinical Lab follows GLP.
4. **Informing the results of screening test:** All the participants will receive the results one week after their screening. Based on the screening results, all participants will be categorized into three groups: (1) infected (HbsAg+), (2) unprotected (HbsAg-/HbsAb-), and (3) protected (HbsAg-/HbsAb+). Dr. Zhiping Li as an Investigator (e.g., Hepatologist) will order all screening tests and review all test results from the certified clinical labs. We will mail the results to participants who are unprotected and

protected. We will not follow up healthy participants who are protected. Dr. Zhiping Li will be responsible for informing the results by calling those infected individuals. Dr. Li will be assisted by other healthcare professionals in the study team including Dr Mark Li (Chinese); Dr. Tran (Vietnamese), and Hepatology Nurse Practitioner, Jae Lee (Korean). They will provide counseling on what the results mean and treatment options. Dr M. Li, Dr. Tran, and Nurse Practitioner,Jae Lee are the consultants in this project.

5. ***F*ollow up for those infected: *T***hose infected can either be referred through their primary care physicians to a hepatologist or directly to a hepatologist. If they do not have a hepatologist, they will be referred to hepatologists in the study team. This will be based on their language preference and geographic location. If they do not have a health insurance, they will be referred to the Asian American Healthcare Center (AAHC), where they will receive free care from a hepatologist. For those infected who are low-income or have no insurance coverage for prescription medication, the study team will provide the information of the Patient Assistance Program run by several pharmaceutical companies including Gilead, GSK, Bristol Myers Squibb, and Schering-Plough.
6. **LHW Intervention/follow up for those unprotected**: Those unprotected will be randomly assigned to either the intervention or the control groups by computer-automated random assignment. Randomization will be used to assure equivalence between groups on key factors that may potentially influence the primary outcome of HBV vaccinations (e.g., gender, age, education, length of stay in the U.S.). The software eliminates the need to do a stratified sampling since it randomly disperses participants with equivalent levels across two groups. If two family members participate in the study with the same result of unprotected, we will ensure that both will be assigned to the same group to prevent contamination.

LHWs will conduct telephone interventions by reminding participants of a series of vaccinations at Months 1, 2, and 6 among those assigned to the intervention group. Those who have health insurance will be encouraged to complete vaccinations through their providers. If they do not have health insurance, LHWs will provide resources to help participants access vaccinations by referring them to the AAHC, or County Health Departments who provide vaccinations to at risk populations. Those in the control group will receive a list of resources by mail that offers free vaccinations, such as local health departments along with their results. Upon completing follow-up, we will provide the delayed LHW intervention for those who do not have vaccinations in the control group.

Seven months after mailing the results, those unprotected will be followed by phone to ask about their status of the series of vaccinations and promoters or barriers to vaccinations. Their self-reported vaccination will be verified with medical records (a copy of prepaid postcard is uploaded). They will be asked to provide information about the date of vaccinations, as well as the location of the clinic or doctor's office where they received vaccinations. They will also be asked to sign a medical release form giving project staff permission to request medical records for their vaccinations. The bilingual interviewers are blinded about participants being in the intervention or control group at post-test.

Data collection will occur at 4 time points

	**Day1**	**Month7**	**Year 1**	**Year 2**
**All participants**	**X**			
**Protected**				
**Unprotected**		**X**		
**Infected**			**X**	**X**
**What to collect**	**Survey, blood sample**	**Telephone interview**	**Interview, blood sample**	**Interview, blood sample**

All clinic activities, education, and medical procedures will be conducted at AAHC, HBI-DC, or JHMI. Study staff will only be involved in collecting data from participants and education. Study staffs will not participate in any clinic administration, medical procedures, or decision-making for the project. The participant contacts that study staffs will have are during the first visit in which the consent form will be explained and questionnaire and

education will be administered, as well as the subsequent telephone follow-up for unprotected. In addition, those infected will be asked the brief questions whether they have medical treatment for their infection.

Analysis will be performed using STATA Version 12. Bivariate and multivariate analyses will be implemented as well as descriptive statatistics.

6. Data Security and Protection of Subject Confidentiality:

Since a large number of participants may not be able to speak English fluently and may be afraid to participate in the study, it is very important that the investigator explain the purpose of the study to them. Consent forms and other written material will be in Asian languages (e.g. Korean, Chinese, and Vietnamese) and will be examined thoroughly for readability.

All participant information will be considered confidential. This confidentiality will be assured through several mechanisms. Each participant will be assigned an anonymous study ID. All the participants will have a separate page for patient identifiers (name, date of birth, telephone number, address, and electronic mail address) to match with the ID. These identifiers will be used for possible follow-up for screening results or vaccinations and will be stored in a locked cabinet accessible only to study staff. Upon completion of this study, all identifiers will be destroyed. All questionnaires and quizzes do not contain any patient identifiers and completed forms will be stored in a locked cabinet accessible only to authorized study staff. The SPSS program will be used for data entry. Upon completion of data entry for an individual participant, we will remove all identifiers (e.g. names and contact information) from the rest of the study data. Moreover, access to all participant data and information will be restricted to authorized personnel. For computerized study data, access will be password protected.

All identifiers and protected health information will be removed from the dataset by authorized study staff for statistical analysis. Authorized personnel will continue to have access to identifiers in order to follow-up with participants regarding their HBV test results or upcoming vaccinations as part of the organization's daily operations. Furthermore, the de-identified dataset will be considered confidential and access to the de-identified dataset will be restricted (password-protected) to authorized personnel only. All data will be reported in aggregate form so that the identity of any individual participant cannot be inferred.

**A. Hard copies of data collection forms:**	
	The study collects data that are anonymous; no personal identifiers are recorded or retained from any study participants in either direct or coded form.
X	Hard copies of data collection materials <u>have identifiers</u> and are locked in a secure cabinet or room with limited access by specified individuals. COPIES WILL BE KEPT IN INVESTIGATOR'S POSSESSION DURING TRANSPORT. When possible, redacted (de-identified) versions of the data collection sheets will be used for coding and analysis.
	Hard copies of data collection materials include an ID code but <u>do not have personal identifiers.</u> However, a code linking the data to the subject's personal information is stored separately from the data collection sheets, and is either stored in a secure electronic database, and/or locked in a secure cabinet or room with limited access by authorized individuals. CODE WILL BE KEPT IN INVESTIGATOR'S POSSESSION DURING TRANSPORT.
	Data are not collected on paper.
	Other (describe):
**B. Electronic Databases:** *Note: This refers to the initial database into which study data is entered and stored. If this "Study Database" includes personal identifiers from participants, only de-identified analytic datasets should be used for data analysis except in instances in which identifying information is required.* ***Databases that retain identifying information require a higher degree of electronic security.***	
	The study collects data that are anonymous; no personal identifiers will be recorded or retained from

	any study participants in either direct or coded form.
X	Personal identifiers are included in the database. If breach of confidentiality poses more than minimal risk to participants because data are personally sensitive in nature (for example, involve substance abuse, mental health, genetic propensities, sexual practices or activities), access to identifiers will be restricted. These data are stored on a secure server protected by strong password, and will be only accessible by authorized study personnel. Data will be coded when possible. Identifiable data transferred or stored via portable electronic devices (e.g., laptops, flashdrives) will be encrypted. The devices on which this information is stored are accessible only to individuals who need access to these data.
	Other (describe):
**C. Analytic Datasets:** *Note: This refers to the use, for analysis, of either discrete subsets or the entirety of the database into which study data is entered and stored. To the extent possible, analytic datasets should be de-identified, except in instances in which identifying information is required.* ***Analytic datasets that retain identifying information require a higher degree of electronic security.***	
	The study collects data that is anonymous; no personal identifiers will be recorded or retained from any study participants.
X	Electronic database will be managed by a specific data administrator (PI or other designated person) who will track and log issuance of analytic datasets, and return/removal when approved use ends. Access to analytic datasets will be subject to conditions established by the PI. Electronic analytic datasets will be provided to authorized study personnel, or approved investigators outside the study, with the same data protection requirements established for the study database.
	Other (describe):

7. Recruitment Process:

We have major channels to recruit participants from the Asian American community.

1) In the past 10 years, the HBI-DC has screened over 3000 individuals in the Baltimore-Washington Metropolitan Area. Thus, many individuals are availing themselves of the free testing services. LHWs will announce upcoming screening events in their community by advertising in Korean churches or Chinese language schools or by word-of-mouth. Our research team and LHWs will attend these events to recruit potential participants.
2) AAHC volunteer clinic is one of the places to recruit participants. Every Saturday, Chinese and Korean patients usually come with family members. If patients and family members are interested in our project, they will be invited to participate in the study after checking eligibility. We do not use their medical records. HBI-DC and AAHC agree to collaborate with this recruitment process and to provide space to conduct the project *(see the attached Memorandum of Understanding and the Budget).*
3) Trained LHWs will announce upcoming screening events in their community by posting flier in Korean churches or Chinese language schools or by word-of-mouth
4) We will identify potential participants by advertising in ethnic media and ethnic groceries (see the flier in the attachment).

8. Consent process and documentation:

Participants will give verbal and written consent before participating in the program. The consent forms will detail the purpose of the study, the requirements for participation, and the potential benefits and risks. We will work with the IRB to include appropriate language and protections in accordance with the data sharing policy. All potential participants will meet with trained study staff fluent in the primary language of the potential participant (Chinese, Korean, or Vietnamese) at either AAHC or one of HBI-DC's health fairs. This staff member will read aloud the consent form to each individual potential participant privately and give the potential participant an opportunity to ask any question before consenting. Participants will be given a copy of the consent form in their native language for their records. Study staff proficient in Chinese, Korean, or Vietnamese

will translate the consent forms, which will be proofread for accuracy of translation by another staff member proficient in that language.

Country(ies)	Consent Document (Indicate “All”, or specify each document when translations vary)	Languages
USA	All	English; Chinese; Korean; Vietnamese

Informing the screening test results: For those infected, the study team will be responsible to inform their infection status and to provide referral list for treatment. The study team will be assisted by other health care professionals who are bilingual consultants in the project. Those infected can be either referred through their primary care physicians to a hepatologist or to a hepatologist directly. If they do not have a hepatologist, they will be referred to the hepatologists associated with the study team. If they do not have a health insurance, they will be referred to the AAHC and Pan Asian Volunteer Health Clinic (PAVHC), community volunteer clinic, where they will receive free care from a hepatologist.

Our study includes two steps of consent procedures. The first one is obtained from all participants when the fieldwork begins in Day 1. The second consent will be annually obtained from Year 1 and 2 from those who found to be chronically infected by the initial screening test for an annual blood test/banking.

(1) For all the participants (Day 1): Based on our previous research experiences with Asian immigrants, we are aware that a large number of these individuals might not have participated in any type of biomedical or behavioral research projects. They may have concerns, fears, or cultural views on providing biospecimen (e.g. blood). Therefore, it is very important that the investigators and bilingual project staffs understand their concerns or cultural views on blood testing and make best efforts to explain them the purpose of the study and potential benefits of the study. Consent forms and other written material will be in Asian-languages along with English (e.g., Korean, Chinese, and Vietnamese) and will be examined for thorough readability. Subjects who wish to participate and pass eligibility screening will be invited to a study. Verification of eligibility and consent will occur in person.

Per National Cancer Institute’s Best Practices for Biospecimen Resources, study personnel (primarily a project staff) will provide detailed explanations on types of hepatitis B tests (e.g., HBsAg, HBsAb, HBcAb), the blood drawing procedure, and the purpose of blood banking (see blood test in the table). The study personnel will make best efforts to explain potential scopes and benefits of future studies in which collected blood samples are used. Testing sites will adhere to standards of Good Clinical Practice and Good Laboratory Practice (GLP) as well as local operating procedures for proper collection, processing, labeling, transport and storage of specimens to ensure integrity of the collected biospecimen. Subjects will be informed about the follow-up procedure specifically, the expectation for future mail and phone contact.

(2) For chronically infected individuals (Year 1 and 2):
Those infected with HBV (HbsAg+) from our initial screening result will be invited to brief interview, follow-up blood testing, and blood banking at JHMI (see the blood test in the table). Participants will receive detailed explanation about the purpose of follow-up, the procedures, and benefits and potential risks about participating in the blood banking. The study personnel will make best efforts to explain potential scopes and benefits of future studies in which collected blood samples are used. They will be explained that they can withdraw the study any time if they wish to do so. If they decide to leave the study, we will arrange their clinic follow-up.

Participants will be asked for their written consent to have their blood samples stored at the research lab for the future study to find the liver cancer biomarkers.

Rationale for biospecimen banking:
Liver cancer is the third leading causes of cancer death worldwide and has a “dismal” outcome, with a 5-year survival rate of only 3% to 5%. The researchers have looked for biomarkers to detect liver cancer early, in the hope that screening could lower the rate of death. There is a great need for tools to diagnosis early, especially among high-risk candidates such as patients with chronic hepatitis B infection. Tumor biomarkers are “attractive potential alternative tools” because they are noninvasive and objective. About 25% of those infected will progress liver cancer. These blood samples are being harvested for developmental purposes. The plasma sample can be used to identify serum biomarkers for hepatocellular carcinoma through a proteomic approach by comparing between the samples prior to and following the development of hepatocelluar carcinoma (HCC) from the same patient. The plasma sample and cryopreserved PBMC can also be used for the analysis of antibodies and antigen-specific T cells that are induced during the carcinogenesis of HCC. Moreover, the cell pellets can be used to analyze the HBV genome and to identify the HBV genomic changes that are associated with the development of HCC. Blood samples from uninfected subjects will be used as controls. When subjects have completed this study, they will have an option of participating in a long-term follow up protocol for the HBsAg+ patients including tests for HBV DNA Quantitative, HBeAg, anti-HBe and liver function panel AFP+L3 and DCP as tumor markers.

Research blood samples will be collected at the time of developing HCC through this long-term follow up protocol. This will be a separate study for the long-term follow up protocol. Due to the nature of developmental studies and the small sample size, no formal statistical analyses are planned. The blood samples will be kept at least for 10 years for developmental studies.

Initial blood sample (n=600), Day 1		Follow-up blood test for those infected (n=60) Year 1 & 2	
HBV screening	Biospecimen Blood sample	Blood test	Biospecimen Blood sample
HBsAg<br>HBsAb<br>HBcAb (anti-HBc)	<u>Serum biomarkers for HCC</u>	HBV DNA quantitative<br>HBeAg<br>Anti-HBe<br>Liver function panel	<u>Serum biomarkers for HCC</u>

9. Risks:

The risks to the patient from blood drawing are very low. Risks include temporary discomfort from a needle stick, bruising, and rarely infection. Lightheadedness may occur but syncope is rare (< 1: 1000). The amount of blood to be drawn is small and of no clinical consequence to the patient. We do not expect any psychological, social, or legal risks.

To minimize the risk from phlebotomy, principal investigator (registered nurse), co-investigators (physicians) or project staff in the field during the study period will be prepared to provide appropriate actions and medical care if any adverse events should arise.

There may also be potential psychological burden (e.g., stigma) for participants who found out that they are infected with HBV. To minimize the psychological impact, our research team will provide a complete explanation and help them follow-up referrals.

10. Benefits
Participants will become aware of hepatitis B virus's transmission mode, potential sequelae, and risk factors associated with HBV from the culturally integrated educational material, Photonovel, which has been developed and tested the importance of HBV screening and improvement of HBV related knowledge from our study of R25. This knowledge will help participants prevent and manage long-term effects of HBV infection. In addition, participants will benefit from knowing their hepatitis B infection status. For those negative but no immunity participants (HBsAg- and anti-HBs -), we will provide resources for free or low cost vaccinations. For those infected, the research team will provide referral recommendations and support to the patients navigate the U.S. health care system (e.g., Patient Assistance Program).

11. Payment:
At the completion of the session, study participants will receive $20 as a token of the appreciation for their time.

12. Drug Products, Vitamins, Dietary Supplements and Devices:
Not Applicable

13. Safety Monitoring:
Not Applicable

14. Plan for reporting unanticipated problems/adverse events
When unanticipated problems or adverse events happened, research staff will notify principle investigator, and then report to IRB immediately.

15. Other IRBs:
The study will be conducted in collaboration with University of Maryland College Park. We will do an IRB authorization agreement for JHBSPH to be the IRB record for University of Maryland College Park once we get the IRB approval from JHBSPH. Its FWA is 00005856.

The contact information of IRB at University of Maryland College Park is as following:
Email: irb@deans.umd.edu
Phone: 301-405-0678
Location: University of Maryland, College Park, 2100 Lee Building, College Park, Maryland 20742

15. Outside collaborations:

The study will be conducted to collaborate with HBI-DC and AAHC for all the data collection procedures including recruitment, informed consent, pretest, education, and HBV screening *(see the attached Memorandum of Understanding).*

Roles and Responsibilities Matrix for IRB Application
Insert Institutions in Collaborator column(s); add additional columns if necessary.

	JHSPH	UMD, College Park	AAHC	HBI-DC
Primary Grant Recipient	X			
Subcontractor		x	X	x

For the following, indicate "P" for "Primary", "S" for "Secondary" as appropriate to role and level of responsibility.) Add additional items if useful.

1	Human subjects research ethics training for data collectors	P	S	S	S
2	Day to day management and supervision of data collection	P	S	S	S
3	Reporting unanticipated problems to the JHSPH IRB/Sponsor	P	S	S	S
4	Hiring/supervising people obtaining informed consent and/or collecting data	P	S	S	S
5	Execution of plan for data security/protection of participant data confidentiality, as described in Sect. 5.	P	S	S	S
6	Biospecimen processing, storage, management, access, and/or future use	P	S	S	S

16. Oversight plan for student studies:

Not applicable

17. Oversight plan for studies conducted at non-JHBSPH sites, including international venues, for which the JHBSPH investigator is the responsible PI:

We will hire and train bilingual research staffs (e.g., Korean, Chinese, and Vietnamese) under two research institutions: JHBSPH and University of Maryland College Park. Research staffs will report to Hee-Soon Juon (Johns Hopkins). All research staffs will need to have a proof of CITI (Collaborative Institutional Training Initiative) training, which is required by JHBSPH Institutional review Board when having contacts with human subjects.

The PI will have a biweekly conference call with investigators and monthly meetings with all study staffs to monitor the progress of the study.

18. Creation of a biospecimen repository:

1) Initial biospecimen banking (Day 1)
The phlebotomist will draw two tubes of blood from study participants at the study site. One tube will be processed for research sample banking which will be transported and stored at Johns Hopkins Medical Institution (JHMI). The other tube will be given the requisition form of HBV screening tests which can be done at JHMI or any certified clinical labs.

For biospecimen banking, we will draw 10 ml peripheral blood into each CPT tube (Becton Dickinson) from each participant. We will have a part-time lab technician to process the blood before banking. Within two hours following blood acquisition, the tubes will be centrifuged at 820*g* for 10 min. The layer of peripheral blood mononuclear cells (PBMC) will be aspirated and centrifuged at 820g for 10 min. The cell pellets will be washed by 10 ml PBS followed by centrifuging at 820g for 10 min. This will be repeated once. The resultant cell pellets will be cryopreserved in liquid nitrogen. Plasma will be aspirated from the remaining sample in the CPT tube and transferred in 1 ml aliquot to each of 1.5 ml tubes, which will be stored at –80°C.

The study team will supervise blood processing and storage for future studies. These blood samples are being harvested. The plasma sample can be used to identify serum biomarkers for HCC through a proteomic approach by comparing the samples prior to and following the development of HCC from the same patient. The plasma sample and cryopreserved PBMC can also be used for the analysis of antibodies and antigen-specific T cells that are induced during the carcinogenesis of HCC. Moreover, the cell pellets can be

used to analyze HBV genome and to identify HBV genomic changes associated with the development of HCC. Research blood samples will be de-identified prior to being used for the developmental studies.

The CPT tube of approximately 10 ml blood will be processed for research sample banking and will be sent to
The Sidney Kimmel Comprehensive Cancer Center and Department of Oncology
Johns Hopkins University School of Medicine
The Bunting-Blaustein Cancer Research Building (CRB1)
1650 Orleans Street, Room 422
Baltimore, MD 21287 (The hard copy of the Biosafety Registration Form was uploaded) The Research Lab will comply with GLP standard of processing and storage.

<u>(2) For chronically infected individuals (Year 1 and 2):</u>
Those infected with HBV (HBsAg+) from our initial screening result will be invited to brief interview, follow-up blood testing, and blood banking at JHMI Year 1 and year 2 after the initial hepatitis B screening. Participants will receive detailed explanation about the purpose of follow-up, the procedures, and benefits and potential risks about participating in the blood banking at the study entry. The study personnel will make best efforts to explain potential scopes and benefits of future studies to prevent liver cancer in which collected blood samples are used (e.g., serum biomarkers for HCC).

19. Data Coordinating Center:

The main data coordinating center will be at the JHBSPH. Principle investigators in both research institutes will constantly check the quality of data collection. Research staffs from each research institute will report to and meet principle investigators weekly. The whole research team from both sites will have face-to-face monthly meetings and conference calls when needed.

Rigorous and systematic quality control will be enforced during all stages of the study. Carefully specified procedures will be adhered to in all aspects of data collection, analysis, and management throughout the study. Data collection includes standardized training and certification of the interviewers, and adherence to carefully specified procedures for interviewing and probing during the interview. The interviewers will be certified after standard training to eliminate interviewer differences; this has also been shown to increase reliability. Data editing will be an important part of each interviewer's job. Each interview will be edited immediately after its completion. Each section of the interview will be reviewed to ensure that all information is complete and accurate. Responses will also be edited to clarify any abbreviations that are not common. Notes concerning the respondent, the interviewing situation and other information potentially useful in the interpretation of the interview will be added during editing. To ensure uniformity of data coding, standardized code rules for entering data will be maintained throughout. The data will then be entered into SPSS program for analysis. As data are received, the records will be logged in and verified for completeness and accuracy of data management. All participants will be assigned a study identification number for tracking purposes.

Data collected will be kept strictly confidential. Individuals will not be identified in comments made during the study or in its publication. Throughout the study, identification of individuals will be solely for scheduling purposes. Once the study database is complete, the identifiers such as name and address will be removed, so that the identification number is the only identifier available. Completed data forms will be placed in locked cabinets. All computerized data will be safeguarded by passwords known only to the authorized personnel.

Contact information for research institutes:

Hee-Soon Juon, PhD
Department of Health, Behavior & Society
Johns Hopkins Bloomberg School of Public Health

624 N Broadway, 704
Baltimore MD 21205
hjuon@jhsph.edu
410-614-5410

Biospecimen banking for those chronically infected: With another consent form to explain the purpose and procedures of follow-up blood tests and blood banking, they will be invited to participate in this follow-up at JHMI in Year 1 and Year 2. Then, their blood samples will be stored at the research lab for future study to find liver cancer biomarker.

Procedures for recruitment, consent, intervention, data collection and analysis will be reviewed and approved by the Committee Human Research at JHBSPH.

Reference

Armstrong, G. L., Wasley, A., Simard, E. P., McQuillan, G. M., Kuhnert, W. L., & Alter, M. J. (2006). The prevalence of hepatitis C virus infection in the united states, 1999 through 2002. *Annals of Internal Medicine, 144*(10), 705-714.

Andersen, M.R. Yasui, Y Meischke, H. Kuniyuki, A. Etzioni, R. Urban, N. (2000). The Effectivness of Mammography Promotion by Volunteers in Rural Communities. *Am J Prev Med*. 18(3):199-207.

CDC. (July 1, 2003). *Hepatitis B vaccination for adults: An evidence-based approach to close the gap in hepatitis B prevention.* Retrieved January 2011 from http://www.strategichealthpolicy.com/portals/0/articles/NVHR_Eliminating_Hepatitis%20-%20Executive_Summary.pdf.

Cohen, J. (1988). *Statistical power analysis for the behavioral sciences*. 2nd ed. Hillsdale, NJ: Erlbaum.

Earp, J. A. L., Viadro, C. I., Vincus, A. A., Altpeter, M., Flax, V., Mayne, L., et al. (1997). Lay health advisors: A strategy for getting the word out about breast cancer. *Health Education and Behavior,24*(4), 432-451.

Hofstede, G. (1991). Cultures and organizations: Software of the mind. New York: McGraw-Hill International.

Hsu, CE., Liu, C.H., Juon H.S., et al. (2007). Reducing liver cancer disparities: a community-based hepatitis B prevention program for Asian-American communities. *Journal of the National Medical Association, 99*, 900-907.

Juon, H. S., Strong, C., Oh, T. H., Castillo, T., Tsai, G., & Oh, L. D. (2008). Public health model for prevention of liver cancer among Asian Americans. *Journal of Community Health, 33*(4), 199-205.

Lefebvre, R. C., & Flora, J. A. (1988). Social marketing and public health intervention. *Health Education Quarterly, 15*(3), 299-315.

Margolis, H. S., Coleman, P. J., Brown, R. E., Mast, E. E., Sheingold, S. H., & Arevalo, J. A. (1995). Prevention of hepatitis B virus transmission by immunization. an economic analysis of current recommendations. *JAMA : The Journal of the American Medical Association, 274*(15), 1201-1208.

Office of Minority Health. (2008). *Chronic hepatitis B in Asian Americans, native Hawaiians and other pacific islanders: Background.* Retrieved April 11, 2009, from http://www.omhrc.gov/templates/content.aspx?ID=7240&lvl=2&lvlid=190#topi
